# Supplementary material for: Circulating Plasma Syncytin-1 mRNA in Preeclampsia—A Pilot Study
Source: Int J Mol Sci. 2025 Sep 16;26(18):9015. doi: 10.3390/ijms26189015 (PMC12470163; doi:10.3390/ijms26189015)
Supplement: Supplementary file 1 [file ijms-26-09015-s001.zip › ijms-3829145-supplementary.pdf]

Isolation of Fetal RNA from Maternal Whole Blood Samples

Samples for the analysis of fetal Syncytin-1 mRNA were collected following a standardized operating procedure established at our institution, using venipuncture and EDTA-coated Monovettes (Sarstedt, Germany) and immediately stored at –80 °C until RNA extraction). Quantification of Syncytin-1 mRNA concentrations was performed *post-partum*; thus, the results had no impact on clinical management or decision-making during pregnancy.

Fetal RNA was isolated from maternal whole blood using the GenUP Virus RNA Kit (biotechrabbit GmbH, Berlin, Germany), following the manufacturer's instructions with minor modifications. For each extraction, a sample volume of 300 µl whole blood was processed. A fresh lysis solution was prepared by mixing 450 µl Buffer Lysis LR with 10 µl Carrier Solution and kept on ice (4 °C) until use. For every extraction, 150 µl thawed whole blood was combined with 450 µl lysis solution and 20 µl Proteinase K. The mixture was vortexed for 10 seconds and incubated at 37 °C for up to 10 minutes to ensure complete lysis. After a brief centrifugation step, 600 µl Binding Buffer BR was added and mixed thoroughly. The lysate was loaded onto spin columns in two steps (650 µl each) and centrifuged at 10,000 g for 1 minute at room temperature. Columns were transferred to fresh collection tubes after each step. RNA-bound columns were washed with 500 µl Wash Buffer A, followed by 650 µl Wash Buffer B, each centrifuged at 10,000 g for 1 minute at room temperature. A final drying step (3 minutes, 10,000 g) ensured removal of residual ethanol. Elution was performed by adding 50 µl of preheated (70 °C) Elution Buffer (ddH_2O), incubating for 2 minutes at room temperature, and centrifuging at 8,000 g for 1 minute. The isolated RNA was stored at –80 °C until further analysis. RNA concentration and purity were assessed using standard spectrophotometric methods.

Reverse Transcription and Pre-amplification

Reverse transcription of total RNA (200 ng) was performed using the FastGene® Scriptase II cDNA Synthesis 5x Ready-Mix (Nippon Genetics, Düren, Germany) according to the manufacturer's instructions. The reaction mixture (20 µL total volume) contained total RNA, 4 µL of 5x Ready-Mix, and RNase-free water to adjust the final volume. The reverse transcription protocol consisted of the following steps: 10 minutes at 25 °C, 60 minutes at 42 °C, and 5 minutes at 85 °C. cDNA products were subsequently stored at –20 °C until further use.

For enhanced sensitivity, the synthesized cDNA was pre-amplified using the TaqMan PreAmp Master Mix Kit (Applied Biosystems, Waltham, MA, USA). The 25 µL pre-amplification reaction contained 12.5 µL of 2x PreAmp Master Mix, 2 µL of custom Syncytin-1 (hSYN1) oligonucleotide primer mix (forward (5′ cttagtgcccccctatgacca 3′) and reverse (5′ gaggttgtgataccgccaat 3′) primers, each at 1 µM), 6.5 µL of nuclease-free water, and 4 µL of undiluted cDNA. The pre-amplification cycling conditions were: enzyme activation at 95 °C for 10 minutes, followed by 10 cycles of denaturation at 95 °C for 15 seconds and annealing/extension at 60 °C for 4 minutes. Pre-amplified cDNA was diluted 1:5 with nuclease-free water prior to downstream quantitative PCR analysis.

Quantitative PCR with Calibration Curve for Absolute Copy Number Determination

Quantitative real-time PCR (qPCR) was performed using the TaqMan Fast Advanced Master Mix (Applied Biosystems). For each reaction, 19 µL of the prepared master mix was combined with 1 µL of the pre-amplified cDNA sample or standard dilution and pipetted in triplicates into MicroAmp® 96-well plates (Applied Biosystems), resulting in a total reaction volume of 20 µL.

The standard curve was generated using the customized SYN1 double-stranded DNA product (dsDNA, gBlocks Gene Fragments synthesized by IDT, Integrated DNA Technologies, Coralville, IA, USA), which corresponds to the amplicon. This standard was serially diluted 1:10 in nuclease-free water, starting from 1×10^11 copies per 3 µL down to concentrations of 1×10^3 copies.

The qPCR reactions were carried out using the 2x TaqMan Fast Advanced Master Mix (Applied Biosystems). For the standard curve and no-template controls (NTC), the master mix consisted of 10 µL of the 2x TaqMan Fast Advanced Master Mix, 3 µL of the standard dilution ranging from 10^7 to 10^3 copies, 1 µL of the Custom TaqMan Gene Expression Assay targeting SYN1 (FAM-labeled, 20x, Applied Biosystems), and 6 µL of nuclease-free water. For sample reactions, 10 µL of the 2x Master Mix was combined with 1 µL of undiluted pre-amplified cDNA, 1 µL of the same Custom TaqMan assay, and 8 µL of nuclease-free water. The qPCR cycling protocol began with uracil-N-glycosylase (UNG) activation at 50 °C for 2 minutes, followed by initial denaturation and polymerase activation at 95 °C for 2 minutes. This was succeeded by 40 cycles consisting of denaturation at 95 °C for 5 seconds and annealing/extension at 60 °C for 25 seconds. Absolute quantification of target gene copies was achieved by interpolating the cycle threshold (Ct) values of the samples against the generated standard curve.
